# Microfluidic device successfully replaces traditional models of pregnancy associated drug pharmacokinetic studies

**DOI:** 10.1002/pmf2.70007

**Published:** 2025-05-01

**Authors:** Ana Collins‐Smith, Pavani Gonnabathula, Miao Li, Sovik Paul, Pilar Flores‐Espinosa, Lauren S. Richardson, Xiaoming Wang, Ramkumar Menon, Ananth Kumar Kammala

**Affiliations:** ^1^ Division of Maternal‐Fetal Medicine Department of Obstetrics & Gynecology The University of Texas Medical Branch at Galveston Galveston Texas USA; ^2^ Division of Critical Care Medicine Department of Anesthesia The University of Texas Medical Branch at Galveston Galveston Texas USA; ^3^ Division of Biochemical Toxicology National Center for Toxicological Research, U.S. FDA Jefferson Arkansas USA; ^4^ Division of Basic Science & Translational Research Department of Obstetrics & Gynecology The University of Texas Medical Branch at Galveston Galveston Texas USA

**Keywords:** GastroPlus, indomethacin, microfluidic systems, PBPK

## Abstract

**Introduction:**

Pregnant and lactating people remain therapeutic orphans as they are often excluded from clinical trials, remaining one of the most therapeutically vulnerable. Current models are not suited to determine pharmacokinetics and dynamics. To mediate this problem, this study evaluated drug propagation using a microphysiologic (MPS) model of the human maternal‐fetal interface (FMi‐PLA‐OOC) with simulation software to see if this could serve as an alternative and more efficient approach to studying pharmacokinetics across the placental barrier during pregnancy surpassing the limitations of traditional placental perfusion systems and animal models.

**Study design:**

The humanized FMi‐PLA‐OOC is composed of seven‐cell culture chambers, connected by microchannels containing umbilical vein endothelial cells, placental cytotrophoblasts and syncytiotrophoblasts, chorion trophoblast cells, amnion mesenchymal and epithelial cells, and maternal decidua cells. A physiological dose of indomethacin (15 µg/mL) was introduced into the decidual chamber to assess drug pharmacokinetics. Media and supernatants of the FMi‐PLA‐OOC were collected at various time points and were analyzed using mass spectrometry to determine the drug concentration. A physiologically based pharmacokinetic (PBPK) pregnancy model was developed using GastroPlus and compared with values from placental perfusion studies, animal models, and clinical data from the literature.

**Results:**

Cells in the FMi‐PLA‐OOC maintained viability, metabolism, and did not show cytotoxicity. The drug propagated and reached the placental layers (umbilical vein cells) in 4 h and the fetal membrane cellular layers (amnion mesenchymal cells) in 1 h. Higher drug concentrations were detected at later time points in the placental cellular layers. Comparing maximum drug concentration ratios in the fetus and mother (*C*_max,fetal_/*C*_max,maternal_) among the different platforms demonstrated: 0.45 (placental perfusion), 0.97 (pregnant humans), 0.774 (FMi‐PLA‐OOC), 0.68 (PBPK). The fold errors of FMi‐PLA‐OOC and PBPK for *C*_max_ ratios compared to placental perfusion were 0.75 and 0.68, demonstrating an acceptable model.

**Conclusion:**

The utilization of the innovative feto‐maternal interface MPS model and PBPK simulation yielded data comparable to the current traditional and simulation approaches. Although not tested here, bedsides kinetics, our humanized MPS model can determine other pharmacologic parameters (efficacy, toxicity, metabolism, absorption, and excretion), making preclinical trials easier, cheaper, and faster and in compliance with the FDA Modernization Act 2.0.

## 1 INTRODUCTION

In the United States, pregnant and lactating people remain therapeutic orphans as they are often excluded from clinical trials for drug development [1]. Pregnant women being classified as therapeutic orphans, they remain one of the most therapeutically vulnerable populations, as evidenced by disease outbreaks and pandemics. To date, if a drug trial includes pregnant patients within its inclusion trial, the investigators have had to demonstrate safety and dosing justification through costly experimental models before the US Food & Drug Administration (US FDA) approval. As an alternative to costly experimental models and advancements in microfluidic technology, numerous organ‐on‐chip (OOC) models have been developed that mimic various organs to study drug transport. Several placenta OOCs have been fabricated. However, most of these placental OOCs fail to include the fetal membranes. To examine both the placental tissue and the fetal interface, under the US DFA Modernization Act 2.0, Richardson et al. and Kamala et al. developed a validated FMi‐PLA‐OOC that looks at the placental‐fetal interface [2, 3, 4]. The FMi‐PLA‐OOC can also be used to determine the known and unknown metabolites of drugs produced by different cells in the placenta and fetal membranes.

The FMi‐PLA‐OOC has provided a more reliable and human‐relevant approach than animal models and a better platform for preclinical drug testing than placenta perfusion [5]. By integrating in silico simulation software such as GastroPlus and Symcyp with relevant physiological parameters and experimental data, we can obtain valuable insights into the pharmacokinetics of drugs during pregnancy. In addition to determining the pharmacokinetics of drugs during pregnancy, the OOC can determine efficacy, metabolism, absorption, and toxicity. This integration of in silico and experimental data offers a practical and efficient means of studying drug behavior in pregnant individuals, holding substantial promise in advancing drug transport research during pregnancy. The scalability of OOCs allows high throughput data generation in minimal time for a thorough analysis of functional and pharmaco‐biological changes in the organs.

Physiologically based pharmacokinetic (PBPK) modeling is vital in understanding drug effects during pregnancy, ensuring maternal and fetal safety by predicting changes in drug disposition [5]. Pregnancy alters physiological parameters, affecting drug absorption, distribution, metabolism, and excretion, necessitating accurate predictive modeling [6]. PBPK models optimize drug dosing regimens, minimize risks like teratogenicity, and aid in drug development by informing clinical trial design and regulatory submissions. They also reduce reliance on animal testing, offering more accurate predictions for human populations while promoting personalized medicine approaches tailored to individual patient characteristics [7]. Overall, PBPK modeling in pregnancy drives advancements in drug safety, dosing optimization, and regulatory processes, ensuring safer and more effective medications for pregnant women.

This study will focus on indomethacin as a prototype drug to evaluate drug propagation & stimulate pregnancy pharmacokinetics. Indomethacin, a non‐steroidal anti‐inflammatory drug (NSAID) that inhibits prostaglandin synthesis, is widely used for managing mild to moderate acute pain, rheumatoid arthritis, and ankylosing spondylitis [8, 9]. Since the 1970s, it has also been used off‐label as a tocolytic agent before 32 weeks of gestation to delay preterm labor by reducing inflammation‐driven uterine contractions [8]. However, despite its widespread clinical use, the FDA has not recommended its use in pregnancy due to concerns about fetal risks, including premature closure of the ductus arteriosus [10] beyond 30 weeks of gestation and the lack of comprehensive pharmacokinetic data to guide dosing and safety assessments [11, 12].

The PBPK of indomethacin in pregnancy has been relatively well characterized in clinical studies and modeling approaches [13]. Indomethacin is known to be highly protein‐bound (90%–99%), weakly acidic, and primarily metabolized by the liver, with significant maternal clearance changes occurring throughout pregnancy. Several studies have assessed its pharmacokinetics in pregnant individuals [11, 14, 15], providing a strong foundation for validating microphysiological system (MPS)‐based and in silico models. Given its clinical relevance, known pharmacokinetic properties, and ongoing consideration for use in managing pregnancy‐associated inflammatory conditions, indomethacin serves as an ideal prototype drug to evaluate drug transfer across the feto‐maternal interface.

Our primary goal is to establish that microfluidic devices combined with in silico simulation models can serve as an alternative method (compared to animal or placental perfusion studies) for determining drug pharmacokinetics during pregnancy. Using indomethacin as a prototype drug, we aim to evaluate its transfer across the feto‐maternal interface and validate the predictive accuracy of these models in comparison to existing clinical and ex vivo data. By integrating MPS with PBPK modeling, we seek to improve drug safety assessments during pregnancy while reducing reliance on traditional in vivo models

## 2 MATERIALS AND METHODS

### 2.1 Institutional review board approval

The study protocol and all related procedures were approved by the Institutional Review Board (IRB) and Institutional Animal Care and Use Committee (ACUC).

### 2.2 Cell preparation and culture

Using previous protocols [3, 16, 17, 18, 19, 20], immortalized primary maternal and fetal cells were collected from term (≥ 37 weeks gestation), not‐in‐labor (TNIL) cesarean deliveries. Cells were cultured with supplements according to Table 1.

**TABLE 1:** Cell types with associated origins, culture media, and supplements.

Cell type	Manufacturer or origin	Culture media	Supplements
Human maternal decidual cells (hFM_DEC)	UTMB TNIL Cesarean delivery	DMEM/F12 (Mediatech Inc., Manassas, VA, United States)	10% FBS, 10% penicillin/streptomycin (Mediatech) 10% amphotericin B (Sigma‐Aldrich, Inc. St. Louis, MO)
Human amnion epithelial cells (hFM_AEC)	UTMB TNIL Cesarean delivery	KSFM	Bovine pituitary extract (30 µg/mL) epidermal growth factor (0.1 ng/mL) CaCl2 (0.4 mM) Primocin (0.5 mg/mL)
Human amnion mesenchymal cells (hFM_ AMC)	UTMB TNIL Cesarean delivery	DMEM/F12	5% FBS 10% penicillin/streptomycin 10% amphotericin B
Human chorion trophoblast cells (hFM_CTC)	UTMB TNIL Cesarean delivery	DMEM/ F12	0.20% FBS 0.01 mM β‐mercaptoethanol 0.5% penicillin/streptomycin 0.3% BSA 1× ITS‐X 2 µM CHIR99021 0.05 µM A83‐01 1.5 µg/mL L‐ascorbic acid 50 ng/mL epithelial growth factor 0.08 mM VPA 1× Revitacell (Rock inhibitor/Y27632).
Primary human umbilical vein endothelial cells (HUVECs)	UTMB TNIL Cesarean delivery	20% FBS	0.1 mg/mL heparin 300 µg/mL endothelial cell growth supplement
Placental cytotrophoblasts (BeWo cells)	ATCC	DMEM/F12 (Mediatech, Manassas, VA, United States)	10% FBS, 10% penicillin/streptomycin (Mediatech Inc.), and 10% amphotericin B (Sigma‐Aldrich).
Placental syncytiotrophoblasts (BeWo cells)	ATCC	DMEM	25 µM forskolin

All cells were grown at 37°C and 5% CO_2_ until they reached 80%–90% confluency. Immortalized cells have been validated against primary cells previously [1, 2, 3, 4, 11, 12]. All cells used in this experiment were under passage twenty‐five.

Primary human umbilical vein endothelial cells (HUVECs) were isolated using previous protocols within the lab at UTMB [17]. In brief, fresh cuts were made on both ends of the umbilical cord and a 21‐gauge needle was inserted into the vein and clamped with a hemostat. A 20cc syringe containing Hanks was attached to the needle and the vein was washed twice with the Hanks. Then, the other end of the vein was clamped, and the syringe was replaced with a new 10 mL syringe containing collagenase. Collagenase was slowly injected into the horizontally placed vein and transferred to a sterile wide beaker filled with PBS to incubate for 30 min at 37°C. Then, the cord was placed in a 50 mL tube to collect loosened cells from the vein and was washed once with Hanks. The collected cells were centrifuged at ∼1200  rpm for 5 min and seeded into a T25 flask. Cells were cultured in a complete medium (noted in Table 1).

As reported in the literature, BeWo cells are used to mimic placental trophoblast cells [21], although this may not be the ideal cell. BeWo cells were chosen for these experiments as no induced pluripotent stem cells or primary cell lines were available to model second or third‐trimester placenta cells.

### 2.3 FMi‐PLA OCC device design, fabrication, cell loading, and experiments

**FIGURE 1:**
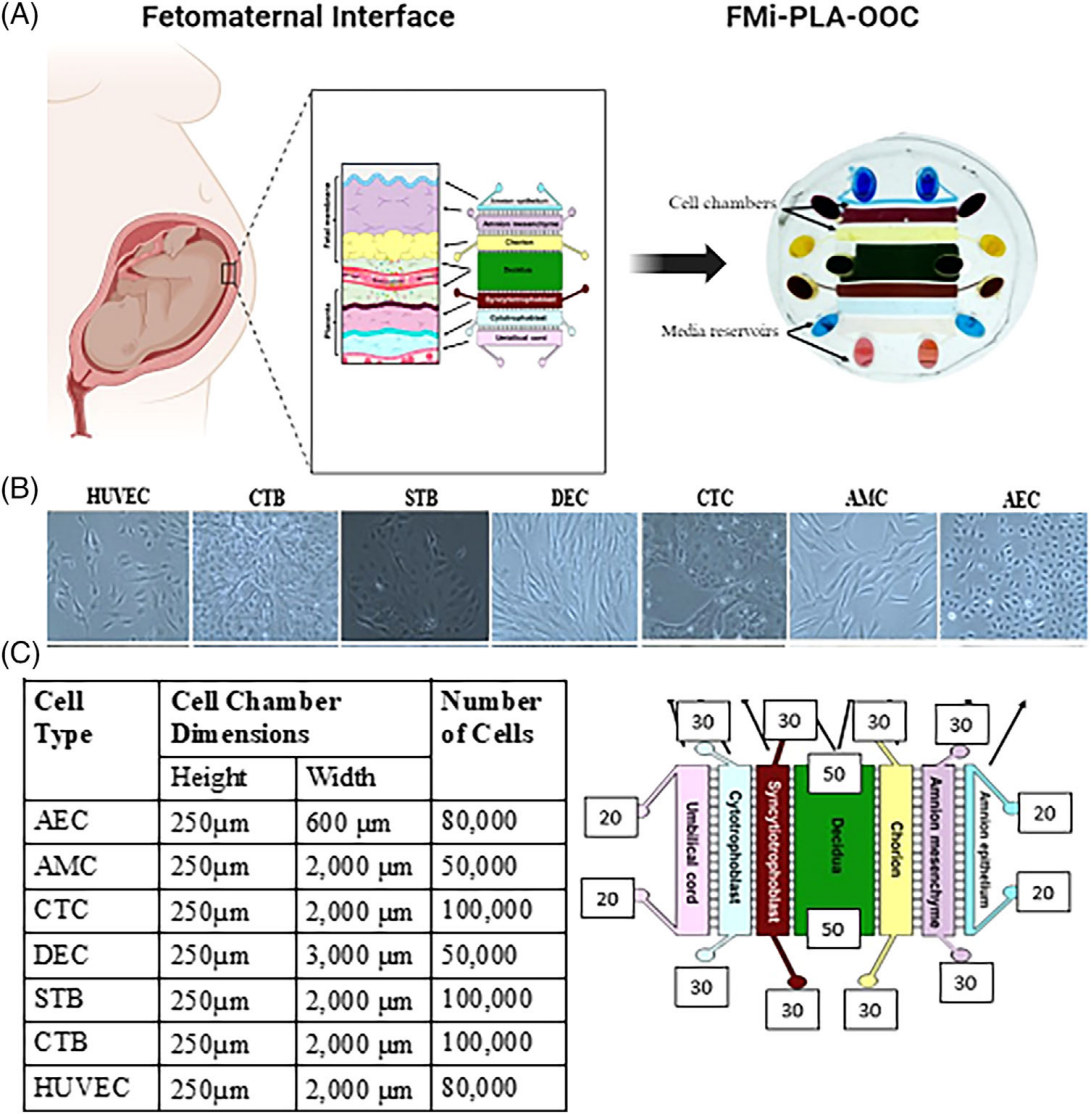
Development of Fetal Membrane‐Placenta Feto‐maternal Interface Organ‐On‐Chip (FMi‐PLA‐OOC) (A) Schematic of feto‐maternal interface corresponding to the FMi‐PLA‐OOC (B) Cell morphology prior to loading on chips (viewed via brightfield microscopy) (C) Cell chamber dimensions (D) Static gradient established with cell‐specific media (E) Characteristics of the microchannels within the FMi‐PLA‐OOC device.

The fetal membrane interfaces (FMis), the fetal membrane, and placenta (Figure 1) play critical roles in nutrient and oxygen transport, paracrine and endocrine signaling between the mother and the baby, perform barrier functions for exogenous substances, and maintain gestational tissue homeostasis [22, 23]. The microfluidic fetal membrane‐placental feto‐maternal interface on‐chip (FMi‐PLA‐OOC) has been previously established through work conducted by Richardson et al. and Kammala et al. [3, 24, 25]. Within the FMi‐PLA‐OOC, fluid diffusion characteristics between cell chambers have been previously established [4] to establish baseline values. The ability to maintain fluidic separation between the seven chambers of the FMi‐PLA‐OOC was tested using Texas Red 3000 KD beads. Due to results from our previous single organ (i.e., FMi‐OOC and PLA‐OOC) and multi‐organ propagation kinetics studies [3, 20, 24], experimental time points of 0.25,1, 2, 4, 8, and 24 h were set to determine indomethacin kinetics across the fetal membrane and placenta FMis.

### 2.4 FMi‐PLA OOC device design

The FMi‐PLA‐OOC is composed of a chip and a reservoir. The chip contains seven rectangular chambers composed of polydimethylsiloxane (PDMS) that form cell cultures resembling the fetal membrane and placenta feto‐maternal interface during gestation (Figure 1a). Each cell chamber is 250  µm in height, and its width was designed to mimic the thickness seen in utero (Figure 1c). Cell chambers were interconnected through an array of 24 microchannels of varying lengths (Figure 1f). The microchannels performed numerous functions, including localized treatment of each cell layer, independent elution of supernatant from each cell chamber, and diffusion between chambers in a time‐dependent manner via active cell migration through cellular transitions. The device also contained an on‐chip reservoir made of PDMS that provided cell media or treatment to the various cells within the FMi‐PLA‐OOC chip. Each reservoir was 2  mm deep and 4  mm in diameter, which aligned on top of the inlets and outlets of each chamber in the primary cell culture layer.

### 2.5 FMi‐PLA OOC device fabrication

The FMi‐PLA‐OOC device was fabricated in PDMS using a two‐step photolithography master mold fabrication process, followed by a soft lithography process of replica molding the final PDMS device from the master mold. A master mold for each chip and reservoir composed of two layers of photosensitive epoxy (SU‐8; MicroChem, Westborough, MA, United States) with different thicknesses was sequentially patterned on a 3‐inch diameter silicon substrate as described in our other chip publications [3, 18, 24, 25, 26]. The master mold was then coated with (tridecafluoro‐1,1,2,2‐tetrahydro octyl) trichlorosilane (United Chemical Technologies) to facilitate PDMS release from the master mold after replication.

The chip and reservoir devices were fabricated from the master molds by pouring PDMS pre‐polymer (Sylgard 184 Silicone Elastomer Base and Sylgard 184 Silicone Elastomer Curing Agent; DowDuPont) onto each in a 10:1 ratio (Base:Curing Agent) followed by curing at 85°C for 45–60 min. After curing, the chips and reservoir devices were carefully removed from their molds. Once the chip was removed from the mold, each inlet was punched using a 1.5 mm punch biopsy needle (Figure 4). Any PDMS obstructing the hole was removed from the reservoir using a 5 mm punch biopsy.

To improve the bonding of the PDMS layer onto the glass substrate and to make the device hydrophilic for easy cell and culture medium loading, the PDMS layers were treated with oxygen plasma (Harrick Plasma) for 120 s, followed by bonding onto a glass substrate (30 mm diameter and 1.5 mm thickness microscope cover slides). This process was repeated to bond the PDMS reservoir layer on top of the device (paying particular attention to line up the reservoir with the chip inlets). After bonding the PDMS reservoir to the chip, the chip–reservoir junction was coated in a thin layer of PDMS, which was then cured at 85°C overnight. The assembled device was then stored dry and sterilized with 70% ethanol for 15 min before use.

### 2.6 FMi‐PLA OOC device collagen and cell loading

Type I (rat tail type I collagen 1:25 serum‐free DMEM/F12 media) and type IV collagen (Corning Matrigel Basement Membrane Matrix, DEV‐free, 1:25 in serum‐free DME/F12 media) were used to fill the microchannels (Figure 1f). Specifically, type I collagen was used to fill the microchannels connecting the HUVECs to CTBs, mimicking the placental basement membrane in utero. Type IV collagen was used to fill the microchannels connecting the AECs to AMCs and AMCs to CTCs, mimicking the amnion and chorion basement membranes in utero. After loading the microchannels and chambers, the devices were incubated at 37°C with 5% CO_2_ for 4 h.

### 2.7 Cell seeding and culture in the FMi‐PLA‐OOC

After incubating the devices at 37°C with 5% CO_2_ for 4 h, a pipette was used to manually remove the collagen from each chamber. The devices were then filled with complete serum‐free DMEM to keep them hydrated and wash out excess collagen. Cells were then loaded onto the device with the cell counts noted in Figure 1c. The devices were placed in a six‐well plate. Each device chamber's reservoir was filled with cell‐specific media to the top of each reservoir. PBS was added to the centers of the six‐well plates to prevent evaporation from the reservoirs. The devices were incubated at 37°C with 5% CO_2_ overnight.

### 2.8 Drug propagation and metabolism across FMi‐PLA‐OCC

A physiological dose of indomethacin (15 µg/mL) [8] was used. Ten milligrams of indomethacin were dissolved in a dimethyl sulfoxide (DMSO) solution. The 10 mg indomethacin/DMSO solution underwent serial dilutions using DMEM/F12 media until the desired 15 µg/mL dose was achieved.

All the media from each reservoir was removed and replaced with fresh cell‐specific media according to the gradient, as noted in Figure 1d. The decidua compartment was the last reservoir and chamber to have its media removed. At this time, indomethacin (15 µg/mL) was added to the decidua chamber and at different timepoints (0.25, 1, 2, 4, 8, 24 h), the supernatant from the different cellular chambers was collected for bioanalytical analysis. The collected supernatants were frozen at ‐20°C until mass spectrometry analysis occurred.

### 2.9 Biological sample preparation and mass spectroscopy analysis

Frozen collected supernatants were thawed at room temperature and then serially diluted with cell‐specific culture media with dilution factors ranging from 1:2.5 to 1:10.

A liquid chromatography in tandem with an electrospray ionization mass spectrometry method was developed and validated for quantification of indomethacin in cell culture medium samples. Indomethacin‐*d_4_* was used as internal standard. Chromatographic separation of indomethacin was achieved on a Waters Symmetry C_18_ HPLC column (100´ 2.1 mm, 3.5 mm) at 45°C. The mobile phase consisted of acetonitrile and water containing 0.05% formic acid (v/v). The flow rate of the gradient elution was 350 µL/min. The API 4000 triple quadrupole mass spectrometer is equipped with a Turbo (V) ion source (ESI) and was operated in positive mode. Multiple reactions monitoring (MRM) mode was applied for the quantification of indomethacin. The MRM transition is *m*/*z* = 344→139 for indomethacin and *m*/*z* = 348→143 for Indomethacin‐*d_4._* The calibration range of indomethacin in cell culture medium samples ranged between 8.79 and 526.5 ng/mL. The samples exceeding the upper limit of quantification were diluted with blank cell culture media for dilution factors ranging from 1:2.5 to 1:1000 depending on the chamber of the FMi‐PLA‐OOC. The accuracy of the method ranged between 93% and 104% with relative standard deviation (RSD) values not exceeding 15%. Quality control samples with high, medium, and low concentration levels were prepared and analyzed along with testing samples to ensure the reliability of the results.

### 2.10 Mass spectrometry protocol untargeted

#### 2.10.1 Instruments and analytical conditions for mass spectrometry analysis

The analysis of indomethacin was performed on an Agilent HPLC 1200 series system coupled with an API 4000 triple quadrupole mass spectrometer (Applied Biosystems). The HPLC system consisted of a degasser (G1379B), binary pump delivery system (G1312A), Hip‐ALS auto‐sampler (G1376B) and column compartment (G1316A) controlled by Analystä1.5 Software (MDS Inc. and Applera Corporation). The indomethacin was separated on Waters Symmetry C_18_ HPLC column (100´2.1 mm, 3.5  mm) connected to a Phenomenex C_18_ guard (4´3.0  mm) at 45°C. The mobile phase consisted of solvent A: acetonitrile (containing 0.05% formic acid, v/v) and solvent B: water (containing 0.05% formic acid, v/v). The flow rate of the mobile phase was 350 µL/min and the elution schedule was 0–3  min, 60%–70% A and then the column with 90% A for 1 min. Before each injection, the HPLC column was re‐equilibrated with 60% A for 5 min. The postcolumn Valco valve (Valco instrument Co. Inc.) was used for directing the elution into the mass spectrometer as following schedule: 0–1.5 min, by‐pass from mass spectrometer; 1.5‐3.0 min, infused into the mass spectrometer and 3.0–9.0 min, by‐pass from mass spectrometer.

The API 4000 triple quadrupole mass spectrometer is equipped with a Turbo (V) ion source (ESI) and was operated in positive mode. Multiple reactions monitoring (MRM) mode was applied for the quantification of indomethacin. The MRM transitions were *m*/*z* = 344→139 for indomethacin and *m*/*z* = 348→143 for Indomethacin‐*d_4._* The source/gas‐dependent MS parameters were as follows: IonSpray Voltage, 5500 V; Curtain Gas, 10 L/h; Ion Source Gas1, 50 L/h; Ion Source Gas2, 25 L/h; Temperature, 450°C; Collision Gas 5 L/h; Interface heater was on. The compound‐dependent parameters for the MRM transition of indomethacin and indomethacin‐*d_4_* were as follows: Declustering Potential, 55 V; Entrance Potential, 9.0 V; Collision Energy, 25 v; Collision Cell Exit Potential, 10 V.

#### 2.10.2 Preparation of stock solution and working standard solutions for mass spectrometry analysis

The stock solution of indomethacin was prepared at 1.5µg/mL in 30% acetonitrile aqueous solution. The working standards solutions were prepared in the range of 35.16–2250 ng/mL. The stock solution of indomethacin‐*d_4_* was prepared at 3.17 µg/mL. The working stock solutions of internal standard (IS) were prepared at final concentrations of 129 ng/mL for indomethacin‐*d_4_*. All stock solutions were stored in −20°C freezer and prepared monthly. All working stock solutions were stored in a 4°C refrigerator and prepared weekly.

#### 2.10.3 Preparation of calibration standards and quality control samples for mass spectrometry analysis

The calibration standards were prepared by spiking 10 µL working stock solutions into 40.0 µL pooled blank cell culture media. The concentrations of calibration standards were 8.79, 17.6, 35.2, 70.3, 140.6, 281.25, 526.5 ng/mL for indomethacin. Quality control samples (QCs) were similarly prepared at high, medium, and low concentration levels for each analyte, as well as for the lower limit of quantification (LLOQ).

#### 2.10.4 Preparation of cell culture media for mass spectrometry analysis

Preparation of cell culture media calibration standards, QCs, and samples was as follows: An IS solution (10 µL) was added to 40.0 µL cell culture media and vortexed for 30 s. Then 100 µL of acetonitrile containing 0.1% (v/v) formic acid solution was added and vortexed for 1 min, followed by centrifugation at 12,000 x *g* for 15 min at room temperature. An aliquot of 70 µL of supernatant was transferred to an HPLC injection vial and 25.0 µL of each sample was injected into the HPLC system.

### 2.11 In silico drug modeling software

#### 2.11.1 GastroPlus

The modeling and simulations were conducted in GastroPlus (version 9.8, Simulations Plus, Inc.), an in‐silico prediction tool for a comprehensive absorption, distribution, metabolism, and elimination (ADME) description [27].

#### 2.11.2 Physiochemical properties used for in silico drug modeling software

**FIGURE 2:**
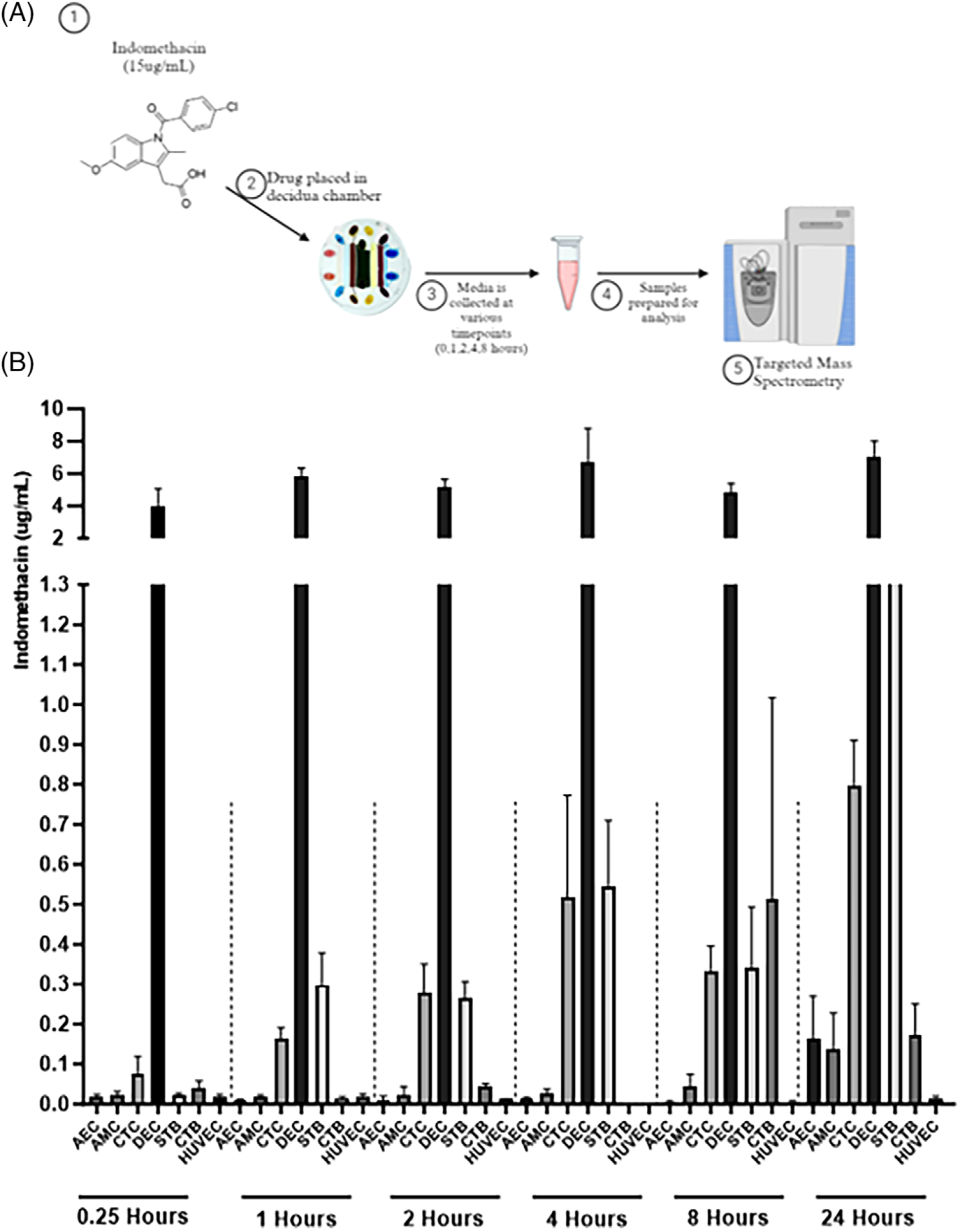
Indomethacin pharmacokinetics across the FMi‐PLA‐OOC (A) Schematic diagram of indomethacin within the decidua chamber of the FMi‐PLA‐OOC (B) Mass spectrometry showed that indomethacin can cross the FMi‐PLA‐OOC within 4 hours and increased over time. Values are expressed as mean intensities ± SEM (N=3).

The chemical structure, physicochemical, biopharmaceutical, and pharmacokinetic parameters of indomethacin were obtained from the literature [9, 11, 13, 14, 15, 28, 29, 30] and incorporated into the software (Figure 2). Based on the chemical structure and ADMET predictor module, we also determined the physio‐chemical parameters that could be compared and refined using the observed data.

#### 2.11.3 Clinical pharmacokinetic data from literature used for in silico drug modeling software

Clinical pharmacokinetic data of indomethacin in pregnant subjects were taken from the work performed by Rytting et al. [11]. Rytting et al. described the mean plasma concentration‐time curve of indomethacin after oral administration of 25 mg every 6 h for a total of five doses. The mean area under the plasma concentration versus time curve at steady state (AUC_ss_) was 1.91 ± 0.53 µg h/mL and the mean peak plasma concentration (*C*_max_) was 1.02 ± 0.49 µg/mL. Placental transfer data of indomethacin were taken from the work performed by Lampela et al. [31] To obtain the values of the mean plasma concentration of indomethacin (from both Lamepla et al. and Rytting et al.), the graphs were scanned with GetData Graph Digitizer (Version 2.26).

**FIGURE 3:**
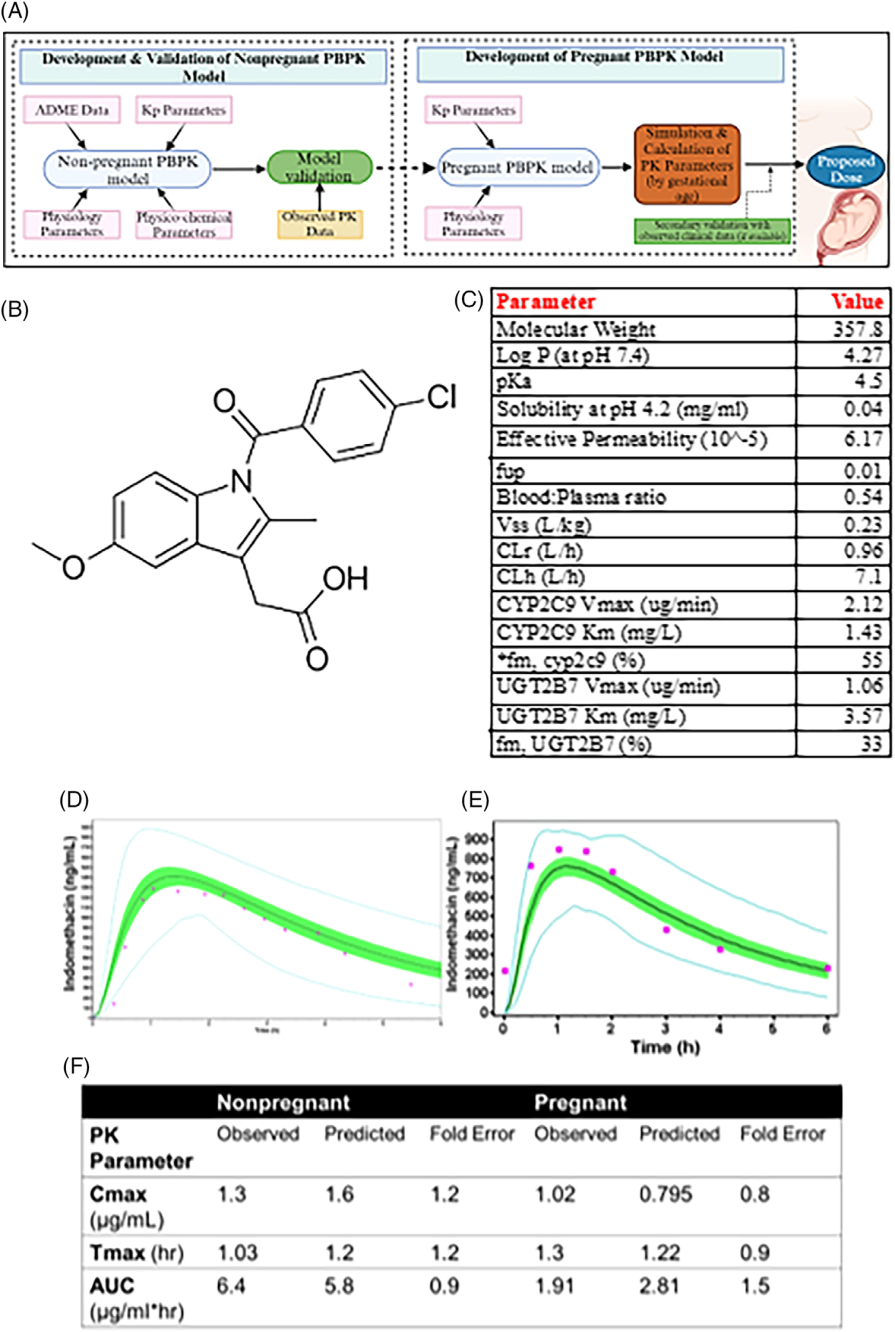
Development & Validation of a Physiologically Based Pharmacokinetic (PBPK) Model in Gastroplus (A) Workflow of developing a pregnancy PBPK in Gastroplus (B) Chemical structure of indomethacin (C) Physicochemical properties of indomethacin (D) Predicted and observed plasma concentrations vs. time profile in healthy non‐pregnant adults (E) Predicted and observed plasma concentrations vs. time profile in pregnant volunteers (N = 25) (F) Predicted and observed pharmacokinetic parameters in both nonpregnant and pregnant models with associated fold errors.

**FIGURE 4:**
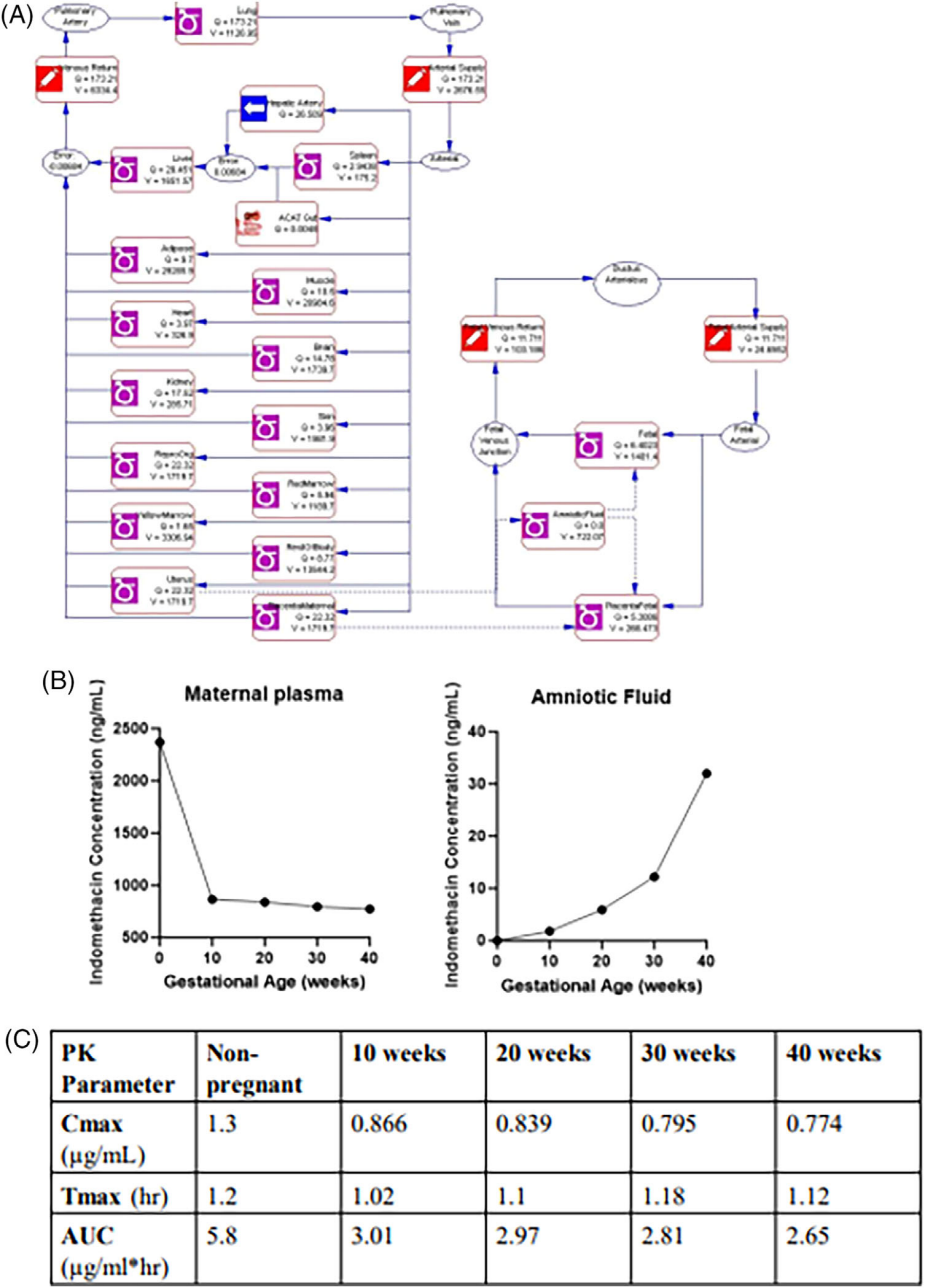
Simulated pregnant pharmacokinetics of indomethacin (A) Structure of PBPK model within Gastroplus (with associated volumes and flow rates) (B) Predicated pharmacokinetics of indomethacin concentrations in maternal plasma and amniotic fluid (C) Simulated pharmacokinetics of indomethacin in the pregnant and non‐pregnant population.

Physiological values for intestinal volumes, lengths, and pH in humans have been built into the software. The stomach transit time was set to 0.35 h. The observed clinical data could be used to develop and validate the PBPK model of indomethacin. The methodology for developing the pregnancy PBPK model is depicted in Figures 3a and 4a.

#### 2.11.4 Development and validation of the indomethacin PBPK model

The development and validation of the physiologically based pharmacokinetic (PBPK) model were divided into three significant steps: (1) establishment of a nonpregnant PBPK model from observed data, (2) validating the developed model with observed data, and (3) applying the validated model to predict the pregnant PBPK model.

To establish a nonpregnant PBPK model, the indomethacin pharmacokinetics data were simulated using the Population Estimated for Age‐Related Physiology (PEAR) module with a size of 25 virtual subjects per population (90% male and 10% female), randomly selected by the software with an average age between 18 and 41 years old. Default values of tissue‐partition coefficients (Kp) were used within in GastroPlus using physiological and compound‐specific determinants of distribution. The refined clearance values incorporated in the software for nonpregnant women were 6.1 and 0.98 ng/mL/min for hepatic and renal excretion, respectively.

The predicted mean values of the PK parameters *C*_max_ (highest plasma concentration), AUC (area under the curve), time at which the drug presents the highest concentration (*T*_max_), volume of distribution (*V*_d_), systemic clearance (CL), and half‐life (*T*_1/2_) were verified with observed data [26]. The developed model was refined by optimizing parameters against clinical data from pregnant subjects and considered validated when there was no deviation ≥5%.

After the nonpregnant model was validated against Olugemo et al. [32] clinical data; a pregnant PPBK model was developed using the aforementioned steps and validated against data from Rytting et al. [11].

### 2.12 Simcyp

#### 2.12.1 Software platform and general model development workflow

**FIGURE 5:**
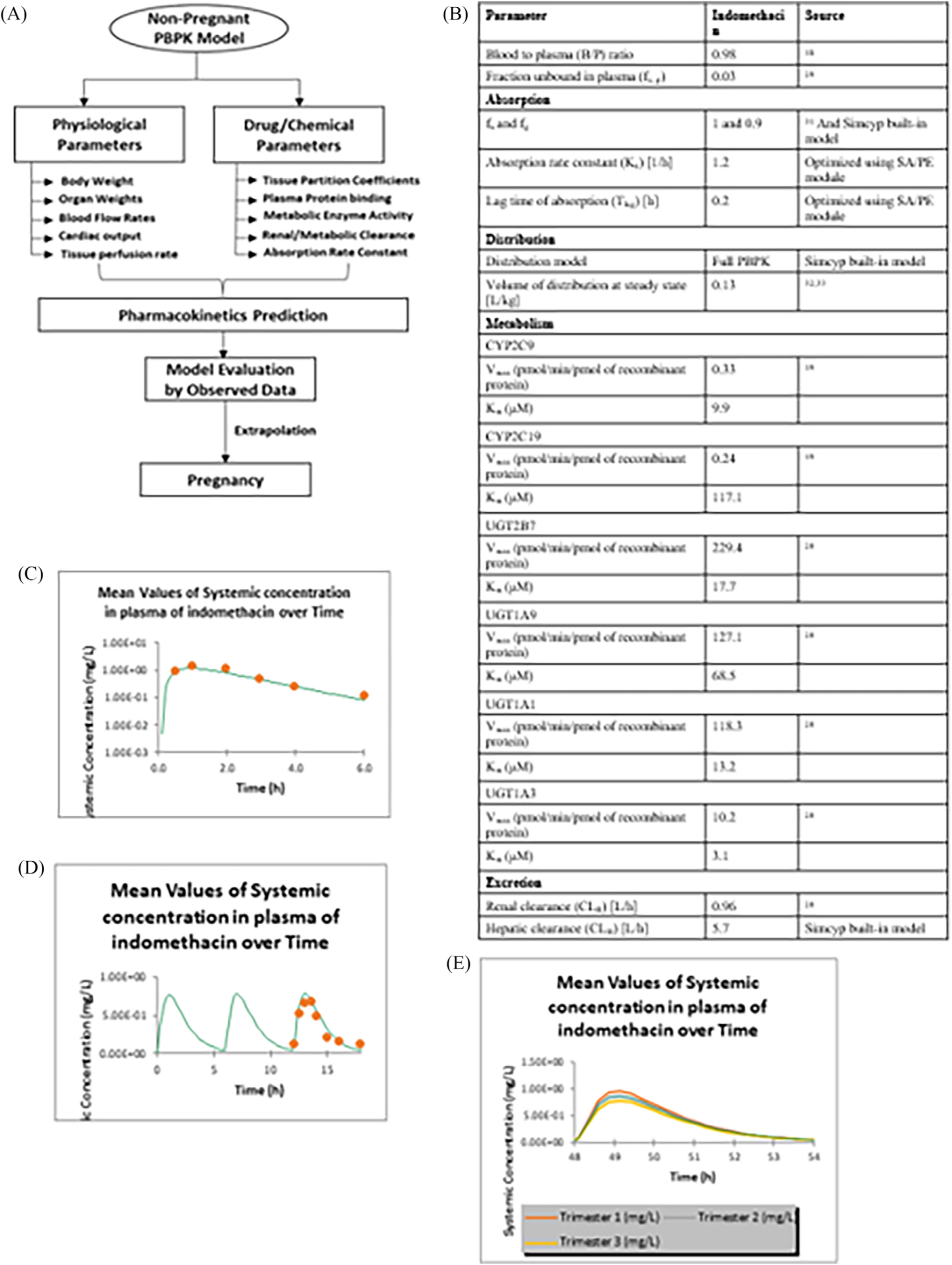
Development & Validation of a Physiologically Based Pharmacokinetic (PBPK) Model in Symcyp (A) Workflow of developing a pregnancy PBPK in Symcyp (B) Physicochemical input properties of indomethacin (C) Predicted and observed plasma concentrations vs. time profile in healthy non‐pregnant adults (D) Predicted and observed plasma concentrations vs. time profile in pregnant volunteers (N=25) (E) Predicted and observed pharmacokinetic parameters in plasma concentrations vs. time profile in pregnant Stratified by trimester.

Indomethacin PBPK modeling and simulations were conducted using Simcyp (version 22, Simcyp Limited, Certara, Sheffield, UK). These simulations incorporated both system‐specific parameters (relevant to the anatomical and physiological characteristics of nonpregnant women, pregnant women, and the fetus) and drug‐specific parameters (including physicochemical properties and ADME characteristics). Initially, PK simulations for indomethacin were carried out in Simcyp's virtual population of healthy nonpregnant individuals. The dose, physicochemical properties, and PK parameters that were used for the indomethacin PBPK model are listed in Figure 5b. The model's predictions for secondary PK parameters such as peak plasma concentrations (*C*_max_), area under the plasma concentration–time curve (AUC), and clearance (CL/F) of indomethacin in nonpregnant women were refined by aligning the model's predicted concentration–time profiles with clinically observed data from nonpregnant subjects [14].

The validated model parameters for indomethacin in nonpregnant individuals were utilized in Simcyp's virtual population of pregnant subjects to forecast PK dynamics during pregnancy. The model's projected concentration–time profiles and secondary PK parameters for indomethacin in pregnant women were then compared against clinically observed data from pregnant individuals to validate the model's accuracy [11]. Model performance was assessed by visually and numerically comparing predicted with observed plasma concentrations and PK parameters. Model performance was considered acceptable if prediction errors did not exceed the twofold error range. The development and validation of the physiologically based pharmacokinetic (PBPK) model workflow are described in a few significant steps (Figure 5).

The OOC system models a critical physiological barrier relevant to drug transport during pregnancy but does not represent the entire maternal‐fetal pharmacokinetic landscape. While the OOC provides experimental data reflective of in vivo barrier function, in silico models can integrate these values into PBPK frameworks, allowing for whole‐system predictions. In this study, we tested two different software platforms to validate the OOC‐derived data, assess the consistency of their predictions, and identify potential limitations in their algorithms. While both approaches provided comparable outputs, discrepancies in predictions also highlighted the need for experimental validation. Our ultimate goal is to develop an integrated OOC‐in‐silico platform that refines PBPK modeling by incorporating empirical micro‐physiological data, enabling more accurate predictions of drug kinetics during pregnancy.

Fold error was calculated to assess the accuracy of simulated pharmacokinetic predictions relative to observed clinical data [33]. This is determined by computing the ratio of the predicted value to the observed value for key pharmacokinetic parameters, such as Cmax and AUC. The formula used for fold error calculation is:

$$\mathrm{Fold}\;\mathrm{Error}=\frac{\mathrm{Predicted}\;\mathrm{value}}{\mathrm{Observed}\;\mathrm{value}}$$

A fold error of ≤2.0 is generally considered acceptable for pharmacokinetic model validation, with a more stringent threshold of ≤1.5 indicating high predictive accuracy.

### 2.13 Statistical analysis

All organ‐on‐chip experiments were conducted in biological triplicates (*n* = 3) using independent chip setups to ensure reproducibility. Mass spectrometry quantifications were performed in technical triplicates for each sample. For PBPK modeling, multiple simulations were conducted to validate model accuracy against clinical pharmacokinetic data. Data was analyzed using Prism 7 (GraphPad, La Jolla, CA, US) software. The Shapiro‐Wilk test was conducted to check for the data's normality. Ordinary one‐way analysis of variance followed by Turkey's multiple comparison tests were used to compare normally distributed data with at least three men. The Kruskal‐Wallis test with Dunn's multiple comparison tests was used for the data that was not normally distributed.

## 3 RESULTS

### 3.1 Indomethacin transfer across the FMi‐PLA‐OOC

To evaluate indomethacin pharmacokinetics across the FMi, a therapeutic concentration of 15 µg/mL [8, 14] (previously determined to be non‐toxic to cells) was introduced into the DEC chamber of the FMi‐PLA‐OOC. Media was collected from each chamber at designated time points and analyzed using targeted mass spectrometry (Figure 2).

Indomethacin reached the AMC chamber (2399 ± 1858.2 ng/mL) and the HUVEC chamber (7.6 ± 19.2 ng/mL), the farthest from the DEC, within 4 h. By 24 h, indomethacin concentrations continued to increase, with a notable rise in HUVECs (15.3 ± 7.3 ng/mL) (Figure 2). These results demonstrate the time‐dependent transfer of indomethacin across the FMi‐PLA‐OOC, providing insight into its transport dynamics within the microphysiological system.

### 3.2 Simulation of drug pharmacokinetics utilizing GastroPlus simulation software

Indomethacin is a weak organic acid that is 90‐99% protein bound to plasma albumin that does not bind to red cells; with properties noted in Figure 3c. The pharmacokinetic properties in a pregnant population at an oral dose of 25 mg every 6 h for five doses included a *C*_max_ of 1.02 ± 0.49 ng/mL and *T*_max_ of 1.3 ± 0.7 h.

Using the physico‐chemical properties of indomethacin, we simulated the reported pregnant pharmacokinetic data using the ADMET predictor module in GastroPlus simulation software. The developed pregnant PBPK model was validated against indomethacin pharmacokinetic studies reported by Rytting et al. [11]. The simulated pharmacokinetic data agreed with the observed values and was within the acceptable fold error of less than 2.0. The observed and simulated indomethacin values are shown in Figure 3f. The pregnancy PBPK model can be seen in Figure 4a. To develop a pregnancy‐related PBPK model, we extended the validated nonpregnant model to modify it for pregnancy‐related pharmacokinetics. GastroPlus software adjusted the intrinsic clearance rates of the fetal and maternal tissues based upon the nonpregnant data.

The concentration‐time profile of indomethacin in pregnancy demonstrated a decrease in the maternal plasma concentration and an increase in the concentration in the amniotic fluid as gestation progressed (Figure 4b). The simulated pharmacokinetic parameters of the pregnant population showed a maximum time (*T*_max_) to reach maximum concentration was 1.2 h, and total drug exposure across time (AUC_0‐t_) was 2.81 µg/mL/h (Figure 3f). The data from GastroPlus simulation demonstrated drug transport across the placenta consistent with placental perfusion studies conducted by Lampela et al.; demonstrating that the simulated model could predict pregnancy pharmacokinetics [31].

### 3.3 Simulation of drug pharmacokinetics utilizing Simcyp software

**FIGURE 6:**
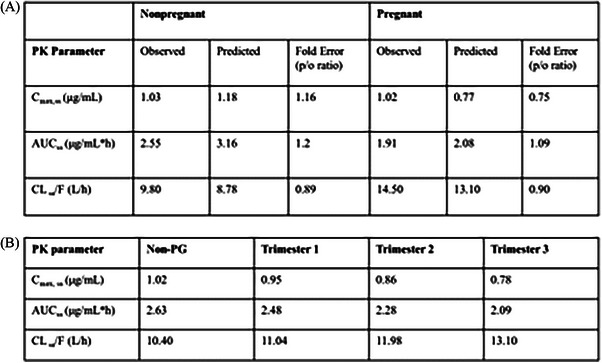
Simulated Pharmacokinetic Parameters in Symcyp (A) Table demonstrating predicted versus observed pharmacokinetic parameters in both nonpregnant & pregnant models with associated fold errors (B) Simulated pharmacokinetic parameters stratified by trimester of pregnancy.

The PBPK‐based prediction for nonpregnant women is validated by comparing the predicted PK parameters with the observed PK parameters of indomethacin in nonpregnant subjects from a clinical study (Figure 6). The ADME model parameters for indomethacin in nonpregnant women were utilized for a Simcyp virtual population of pregnant women [13]. The predicted PK profiles of indomethacin are comparable to clinical data in pregnant women. Model prediction of indomethacin plasma concentration versus time profiles was in good agreement with the observed clinical data. The fold errors in the prediction of *C*_max_ and AUC of indomethacin in pregnant women were 0.75‐fold and 1.09‐fold, respectively (Figure 6), which is also comparable with the GastroPlus prediction.

### 3.4 Comparison of results from FMi‐PLA‐OOC and simulation model

We compared values from placental perfusion studies, animal models, and clinical data from the literature to our in‐silico simulation model and FMi‐PLA‐OOC data to validate and establish the biological relevance of drug transfer rates. In the placental perfusion model, indomethacin demonstrated a transfer rate of 15 ± 5%. Compared to the FMi‐PLA‐OOC, the percentage of indomethacin transfer to the fetal side was 15.1%. In simulation software, there was a 14% transfer rate, and in human studies, there was a 25% transfer rate. The observed ∼10% difference between FMi‐PLA‐OOC, PBPK simulations, and human studies is likely due to the MPS modeling a controlled placental barrier in isolation, whereas human pharmacokinetic data reflect the complex interplay of systemic circulation, placental metabolism, and individual variability. While this difference may suggest a slight underestimation, it remains within an acceptable range for preclinical models, supporting the utility of integrating OOC data into PBPK frameworks for refined predictions of drug transfer during pregnancy.

## 4 COMMENT

Currently, 100% of the worldwide population would not exist without a pregnant person. Despite this unique fact, pregnant and lactating people remain therapeutic orphans due to the sole exclusion criterion of being pregnant. As pregnant people are excluded from clinical drug development and therapeutic trials, there is a lack of knowledge about drug pharmacokinetics during pregnancy. To address this problem, we evaluated drug propagation using an MPS model of the human maternal‐fetal interface (FMi‐PLA‐OOC) with simulation software & validated it to determine that it could serve as an alternative and more efficient approach to studying pharmacokinetics across the placental barrier during pregnancy. This study focused on comparing indomethacin transfer rates within in vitro FMi‐PLA‐OOC, in silico GastroPlus, in silico Symcyp, ex vivo placental perfusion, and current clinical data.

As indomethacin is currently being used off‐label for tocolysis in the setting of preterm labor in pregnancies less than 32 weeks’ gestation, providing its rate of transfer and metabolism across both the fetal‐maternal interfaces is valuable to clinicians as there have been reports of maternal indomethacin use associated with poor fetal outcomes such as oligohydramnios, pulmonary hypertension, and gastrointestinal perforation. This could be supported by the simulated data demonstrating a sharp increase in the concentration of indomethacin in the amniotic fluid noted around 30 weeks of gestation.

**FIGURE 7:**
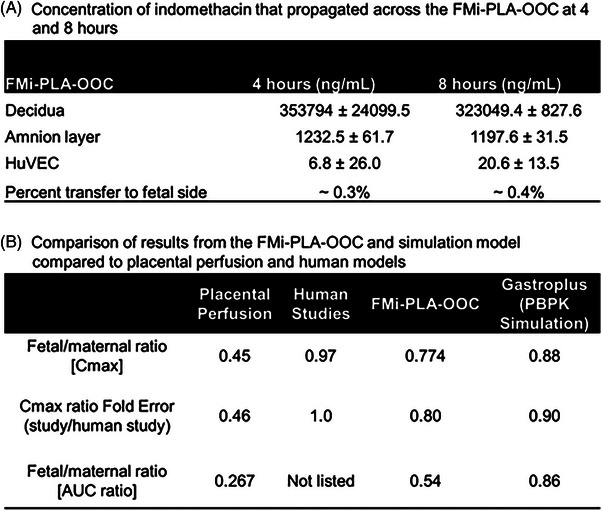
Validation of the FMi‐PLA‐OOC (A) Concentration of indomethacin that propagates across the FMi‐PLA‐OOC at 4 and 8 hours in the amnion and epithelial cells (B) Comparison of results from the FMi‐PLA‐OOC and simulation model compared to placental perfusion and human models.

Within the FMi‐PLA‐OOC we document the pharmacokinetics of indomethacin across the feto‐maternal interface. We have shown that OOCs can be used to study drug pharmacokinetics in pregnancy as the transfer rates of indomethacin within the OOC are comparable to placental perfusion studies, simulation software, and human studies (Figure 7).

Not only have we demonstrated indomethacin pharmacokinetics across OOCs, but we have also demonstrated simulation software can accurately predict pharmacokinetics, as noted with the use of two different simulation software. Within the pregnant PBPK models, pharmacokinetic parameters indicate that indomethacin exhibits approximately 6, 7, 15, and 25% higher clearance and 48, 48, 51, and 54% lower exposures in pregnant women from 10, 20, 30, and 40 weeks’ gestation. This suggests the need for dosage modification of indomethacin for different gestational ages.

As the pregnancy progresses, the fetal exposure increases as the amniotic fluid concentration of indomethacin increases. Physiological changes noted in pregnancy support lower maternal plasma concentrations and higher maternal clearance.

The simulations produced by the two different software platforms correlate with each other and have fold errors less than 1.5. A fold error of less than 1.5 is considered an acceptable simulation model. As both the simulation software and OOCs demonstrate an equivocal transfer rate, this demonstrates the utility of OOC devices for drug research in pregnancy.

Our findings demonstrate that the integration of MPS and PBPK modeling provides a reliable approach for evaluating drug transfer across the feto‐maternal interface. The observed indomethacin transfer rates in the FMi‐PLA‐OOC (15.1%) and PBPK simulations (14%) closely aligned with ex vivo placental perfusion studies (15 ± 5%), reinforcing the physiological relevance of our platform. However, the 10% difference between these models and human clinical data (25%) likely stems from the controlled nature of the OOC system, which isolates the placental barrier and does not account for systemic circulation, placental metabolism, or interindividual variability present in vivo. While this discrepancy may suggest a slight underestimation of drug transfer, it remains within an acceptable range for preclinical models, underscoring the potential of MPS‐PBPK integration in refining pregnancy pharmacokinetic predictions.

Some strengths of this study include using both fetal and placental membranes in the organ‐on‐chip to mimic in‐vitro drug transporters [21, 22, 34, 35, 36, 37, 38, 39, 40], the ability to conduct large‐scale studies, the ability to mimic physiological conditions, and integration with simulation software. An additional strength of this study was that we used two different simulation software to model the pharmacokinetics of indomethacin in pregnancy. Another strength unique to this study is that it examined the effects of varying gestational age (across all three trimesters) on the pharmacokinetics of indomethacin.

The main limitation of this study is that simulation software to conduct PBPK modeling is not yet widely available and is expensive. Additional limitations of this study include the use of a novel FMi‐PLA‐OOC device that is not widely available, the use of TNL placentas, subject variability of placental cellularity, and a static FMi‐PLA‐OOC model.

## 5 CONCLUSION

The integration of microfluidic OOC technology with simulation software provides a human‐relevant, cost‐effective, and physiologically adaptable approach to studying drug behavior during pregnancy. This platform not only enables the screening of novel therapeutics for pregnancy‐related conditions but also facilitates the evaluation of over‐the‐counter (OTC) drugs that are commonly used during pregnancy, addressing a critical gap in maternal‐fetal pharmacology. Furthermore, this approach aligns with the principles of the US FDA Modernization Act 2.0 by promoting the refinement and reduction of animal studies while providing a more scalable and ethical alternative for drug testing. By integrating empirical OOC‐generated data within silico models, this system offers a powerful tool for improving drug safety assessments and optimizing pharmacotherapy for pregnant individuals.

## DISCLOSURE

This article reflects the author's views only and does not necessarily represent those of the U.S. Food and Drug Administration.

## Supporting information



Supporting Information

## Data Availability

Available upon request from the corresponding author.
