# Supplementary material for: Microfluidic device successfully replaces traditional models of pregnancy associated drug pharmacokinetic studies
Source: Pregnancy (Hoboken). 2025 May 1;1(3):e70007. doi: 10.1002/pmf2.70007 (PMC13344748; doi:10.1002/pmf2.70007)
Supplement: Supplementary file 1 [file PMF2-1-e70007-s001.docx]

| **Parameter** | **Indomethacin** | **Source** |
| --- | --- | --- |
| Molecular weight (g/mol) | 357.8 | ^17^ |
| Log P | 3.53 | ^17^ |
| Compound type | Monoprotic acid | ^17^ |
| pKa | 4.5 | ^17^ |
| Blood to plasma (B/P) ratio | 0.98 | ^18^ |
| Fraction unbound in plasma (f_u, p_) | 0.03 | ^29^ |
| **Absorption** | | |
| Absorption model | First order | Simcyp built-in model |
| f_a_ and f_g_ | 1 and 0.9 | ^31^ And Simcyp built-in model |
| Absorption rate constant (K_a_) [1/h] | 1.2 | Optimized using SA/PE module |
| Lag time of absorption (T_lag_) [h] | 0.2 | Optimized using SA/PE module |
| **Distribution** | | |
| Distribution model | Full PBPK | Simcyp built-in model |
| Volume of distribution at steady state [L/kg] | 0.13 | ^32,33^ |
| K_p_ scalar | 1 |  |
| **Metabolism** | | |
| CYP2C9 | | |
| V_max_ (pmol/min/pmol of recombinant protein) | 0.33 | ^19^ |
| K_m_ (μM) | 9.9 |  |
| CYP2C19 | | |
| V_max_ (pmol/min/pmol of recombinant protein) | 0.24 | ^19^ |
| K_m_ (μM) | 117.1 |  |
| UGT2B7 | | |
| V_max_ (pmol/min/pmol of recombinant protein) | 229.4 | ^20^ |
| K_m_ (μM) | 17.7 |  |
| UGT1A9 | | |
| V_max_ (pmol/min/pmol of recombinant protein) | 127.1 | ^20^ |
| K_m_ (μM) | 68.5 |  |
| UGT1A1 | | |
| V_max_ (pmol/min/pmol of recombinant protein) | 118.3 | ^20^ |
| K_m_ (μM) | 13.2 |  |
| UGT1A3 | | |
| V_max_ (pmol/min/pmol of recombinant protein) | 10.2 | ^20^ |
| K_m_ (μM) | 3.1 |  |
| **Excretion** | | |
| Renal clearance (CL_R_) [L/h] | 0.96 | ^20^ |
| Hepatic clearance (CL_H_) [L/h] | 5.7 | Simcyp built-in model |

**SUPPLEMENTARY MATERIAL:**
